# Differential psychological response to the COVID-19 pandemic in psychiatric inpatients compared to a non-clinical population from Germany

**DOI:** 10.1007/s00406-021-01291-7

**Published:** 2021-07-15

**Authors:** Stephanie V. Rek, Daniel Freeman, Matthias A. Reinhard, Markus Bühner, Sofie Grosen, Peter Falkai, Kristina Adorjan, Frank Padberg

**Affiliations:** 1 Department of Psychiatry and Psychotherapy, LMU University Hospital Munich, Nussbaumstraße 7, Munich, Germany; 2 International Max Planck Research School for Translational Psychiatry (IMPRS-TP), Munich, Germany; 3 Department of Psychiatry, University of Oxford, Oxford, UK; 4 Department of Psychology, LMU Munich, Munich, Germany

**Keywords:** COVID-19 pandemic, Mental health, Psychiatric inpatients, COVID-19-specific stressors, Psychological response

## Abstract

The COVID-19 pandemic is an inherently stressful situation, which may lead to adverse psychosocial outcomes in various populations. Yet, individuals may not be affected equally by stressors posed by the pandemic and those with pre-existing mental disorders could be particularly vulnerable. To test this hypothesis, we assessed the psychological response to the pandemic in a case–control design. We used an age-, sex- and employment status-matched case–control sample (*n* = 216) of psychiatric inpatients, recruited from the LMU Psychiatry Biobank Munich study and non-clinical individuals from the general population. Participants completed validated self-report measures on stress, anxiety, depression, paranoia, rumination, loneliness, well-being, resilience, and a newly developed index of stressors associated with the COVID-19 pandemic. Multiple linear regression analyses were conducted to assess the effects of group, COVID-19-specific stressors, and their interaction on the different psychosocial outcomes. While psychiatric inpatients reported larger mental health difficulties overall, the impact of COVID-19-specific stressors was lower in patients and not associated with worse psychological functioning compared to non-clinical individuals. In contrast, depressive symptoms, rumination, loneliness, and well-being were more strongly associated with COVID-19-specific stressors in non-clinical individuals and similar to the severity of inpatients for those who experienced the greatest COVID-19-specific stressor impact Contrary to expectations, the psychological response to the pandemic may not be worse in psychiatric inpatients compared to non-clinical individuals. Yet, individuals from the general population, who were hit hardest by the pandemic, should be monitored and may be in need of mental health prevention and treatment efforts.

**Supplementary Information:**

The online version contains supplementary material available at 10.1007/s00406-021-01291-7.

## Introduction

Many unprecedented stressors caused by the COVID-19 pandemic may contribute to increased psychological and emotional distress, reduced levels of well-being, and thus pose a substantial risk for an emerging mental health crisis worldwide [1]. While COVID-19 itself represents an obvious threat for physical health and imposes burden on individuals and groups worldwide, numerous stressors are also resulting from the politically enforced restrictions (e.g., stay-at-home orders) and recommended behaviours (e.g., physical distancing, often referred to as social distancing) to minimize face-to-face interactions. Although these may partly be latent changes to people’s lives, these pandemic-related restrictions may have a profound and long-lasting societal and economic impact on many individuals due to infringement, for example, on personal freedoms, uncertainty and concern over disease status, social isolation, job uncertainties, and financial hardship.

As vulnerability to psychosocial stressors varies, some individuals may be more affected by the adverse impact of the COVID-19 pandemic than others. According to the diathesis-stress-model [2] of mental disease, individual differences are thought to arise from a complex interplay between pre-existing risk factors (diatheses) and current environmental stressors. As such, environmental stressors may exert their most pronounced negative effects on mental health in vulnerable individuals with a specific genetic makeup and pre-existing mental health difficulties. This framework has been pioneered in the context of smoking [3] and has since been applied for a variety of other mental health disorders [4, 5]. In line with this theory, the psychological response to the pandemic should theoretically be greatest for vulnerable individuals with severe mental health disorders as has been predicted by several recent scientific publications [6–8]. Yet, it remains unclear if psychiatric patients experience more psychiatric symptoms specifically due to the COVID-19 pandemic. Addressing this key question is clinically relevant. It could help to identify individuals with the greatest mental health needs, develop appropriate mitigation strategies for managing the psychological consequences of the COVID-19 pandemic, and safeguard vulnerable individuals who were hit hardest by the pandemic.

The pandemic’s psychological impact on patients with severe mental disorders remains largely unknown. Previous epidemics and pandemics have led to increased mental health difficulties [9, 10] and preliminary epidemiological studies and meta-analyses have quantified psychiatric symptom prevalence in the COVID-19 pandemic [11, 12]. In the general population, a prevalence of 29.6% for stress symptoms, 31.9% for anxiety symptoms, and 33.7% for depressive symptoms have been reported [12]. In such meta-analytic work, however, prevalence rates have not been interpreted in the context of symptom prevalence rates prior to the pandemic, which obfuscates inferences about the actual mental health impact of the pandemic. Initial longitudinal research comparing mental health difficulties before and during the pandemic describe an increase in mental health difficulties (e.g., anxiety, depression, stress, suicide risk, & post-traumatic stress) during the early stages of the pandemic using data from the UK Household Longitudinal Study panel [13]. In addition, a recent meta-analysis highlights a modest but consistent mental health impact of COVID-19 lockdown measures, particularly for depressive and anxiety symptoms [14]. Yet, the longer-term effects remain unknown and it is unclear if the pandemic has a particularly pronounced impact on the mental health of psychiatric patients. While initial case–control studies have found general differences in symptom prevalence rates as would be expected [15, 16], Pan et al. [17] also provided longitudinal mental health comparisons before versus during the early stages of the pandemic and using COVID-19-specific items beyond general symptom questionnaires in three large Dutch case–control cohorts. Interestingly, the authors demonstrate that patients’ mental health functioning was similar before versus during the early stages of the pandemic, while healthy individuals experienced more symptoms during compared to before the pandemic. The authors offer several explanations of these findings including mitigation strategy-induced relaxation, feelings of safety, or simply regression to the mean. Similarly, a more recent longitudinal population-based study conducted in the United States showed a sharp initial increase in psychological distress in individuals with pre-existing mental health conditions during the early phases of the pandemic (April 2020). However, distress levels decreased to baseline levels in the weeks that followed (July 2020) highlighting the potential role of resilience in the psychological response to the pandemic [18]. Similar results were also observed by other research groups [19–22] and summarised in a systematic literature review of population-based longitudinal cohort studies [23]. The impact of COVID-19-specific stressors could offer an additional explanation, which can only be studied using a more fine-grained dissection of the pandemic’s psychological response.

In the present study, we investigated the impact of COVID-19-specific stressors on a diverse range of psychosocial outcomes using validated self-report measurement scales in a case–control comparison matched on age, sex, and employment status and using the COVID-19-specific stressor impact index of the newly developed COVID-19 Pandemic Mental Health Questionnaire (CoPaQ) [24]. In line with the diathesis-stress-model, we hypothesised that psychiatric inpatients are more negatively affected by COVID-19-specific stressors compared to non-clinical controls from the general German population in terms of higher levels of anxiety, depression, stress, paranoia, rumination, and loneliness as well as lower levels of well-being and resilience.

## Methods

### Participants

#### Clinical sample

The clinical sample (*n* = 108) was recruited as part of the LMU Biobank study and was composed of psychiatric inpatients from the Department of Psychiatry and Psychotherapy of the LMU University Hospital Munich. Participants indicated demographic information and filled out self-report questionnaires (order: CoPaQ, DASS-21, R-GPTS, WHO-5, UCLA, SNI, & BRS) using paper–pencil. Psychiatric inpatients with insufficient comprehension of German, an acute psychotic or manic episode, or acute suicidality were excluded from participation.

#### Non-clinical sample

The non-clinical control sample was recruited online from the general German population using advertisements on social media (Facebook) and via university mailing lists. Assessments were made via a secure online survey software (LimeSurvey). This study is part of an ongoing longitudinal survey into the mental health consequences of the pandemic. The non-clinical sample completed the same questionnaire batterie, which was presented in a block randomised order to reduce carry-over effects and using a forced response format. At the end, participants were asked to enter their email address to be included in a prize draw. The sample consisted of adults (18+ years). In total, 387 (77.87%) identified as women, 108 (21.73%) as men, and 2 (0.40%) as diverse with an age range from 18 to 75 years (mean = 30, standard deviation (SD) = 11).

#### Matching

To obtain a more comparable case–control sample in terms of key sociodemographic factors, the clinical and non-clinical samples were matched on age, sex, and employment status using *R* software and the *MatchIt* (v4.1.0) package [25]. Matching is preferable over sole adjustment of potential confounders in regression analyses since it increases sample comparability and efficiency of analyses as similar numbers of cases and controls are present across confounder strata [26]. After matching, clinical and non-clinical samples were comparable in age and sex (age: t(212.56) = − 1.47, *p* = 0.142; sex: *χ*^*2*^(1) = 0.07, *p* = 0.785), but differences remained for employment status (*χ*^*2*^(6) = 27.22, *p* < 0.001).

#### Ethical approval and informed consent

The study was subject to ethics committee approval (clinical sample [Project Number: 18-716]; non-clinical sample [Project Number: 20-118]) and conducted in accordance with the Declaration of Helsinki [27]. All participants provided informed consent. Recruitment in both study groups took place between April-December 2020.

### Data integrity and quality control

Integrity of participants’ responses and data was ascertained in multiple pre-processing steps (see Supplementary Methods and Supplementary Fig. 1 for an overview).

### Measures

#### COVID-19 Pandemic Mental Health Questionnaire (CoPaQ)

The CoPaQ (https://osf.io/3evn9/) [24] is a newly developed and highly comprehensive self-report measure assessing the psychosocial impact of the COVID-19 pandemic. For the purpose of this study, we included data of an index assessing the impact of COVID-19-specific stressors over the past 2 weeks from the CoPaQ. Individual stressors included among others quarantine/curfew, small accommodation/home-office, financial difficulties, childcare responsibilities, and physical health concerns; we provide a full list of items in Table 1 and Supplementary Fig. 2 depicts COVID-19-specific stressors inter-item correlations. Each stressor was rated using a 5-point Likert scale ranging from 0 (Not at all) to 4 (Very much) and participants’ responses of “Not applicable” were recoded as 0. A sum score of all items was calculated as an index of COVID-19-specific stressors with higher scores indicating a greater stressor impact. We observed an acceptable internal consistency of the COVID-19-specific stressors scores with McDonald’s Omega (*ω)* = 0.79 (95% confidence interval [CI]: 0.75–0.84). It is important to note, however, that stressors are likely to occur relatively independently, so a high internal consistency was not necessarily presumed.

**Table 1:** Socio-demographics and baseline characteristics of the matched clinical and non-clinical samples

	Clinical sample	Non-clinical sample
Age, mean* (SD)*	43.97 (14.71)	41.14 (13.54)
Women sex, *n (%)*	54 (50.00%)	51 (47.22%)
Employment status, *n (%)*		
Full-time employed	32 (29.63)	50 (46.30)
Part-time employed	17 (12.96)	14 (15.74)
Self-employed	15 (4.63)	5 (13.89)
Student	7 (6.48)	7 (6.48)
Retired	5 (16.67)	18 (4.63)
Caregiver	0 (0)	0 (0)
Not employed	24 (22.22)	14 (12.96)
Other	8 (7.41)	0 (0)
Essential activity for the maintenance of critical infrastructure, *n (%)*		
Doctors	1 (0.9)	2 (1.9)
Nurses	3 (2.8)	7 (6.5)
Clinical psychologist	0 (0)	1 (0.9)
Public safety and national security guards	0 (0)	1 (0.9)
Staff of local and national government	0 (0)	1 (0.9)
Supermarket vendors	2 (.9)	0 (0)
Professional cleaners	1 (0.9)	1 (0.9)
Other (not listed)	20 (18.5)	15 (13.9)
No	81 (75.0)	80 (74.1)
Self-reported lifetime diagnoses, *n (%)*		
Number of diagnoses		
0	0 (0)	71 (65.74)
1	29 (26.85)	18 (16.67)
2	37 (34.26)	12 (11.11)
3	24 (22.22)	7 (6.48)
> = 4	18 (16.67)	0 (0)
Any diagnosis	108 (100)	37 (34.26)
Diagnostic categories		
Depressive disorders	88 (81.48)	30 (27.78)
Bipolar disorders	10 (9.26)	2 (1.85)
Psychotic disorders	17 (15.74)	1 (0.93)
Anxiety disorders	30 (27.78)	14 (12.96)
Post-traumatic stress disorder	17 (15.74)	2 (1.85)
Obsessive–compulsive and related disorders	6 (5.56)	1 (0.93)
Disorders		
Eating disorders	17 (15.74)	3 (2.78)
Substance-related and addictive disorders	30 (27.78)	4 (3.70)
Attention-deficit/hyperactivity disorder	6 (5.56)	3 (2.78)
Somatoform disorders	7 (6.48)	2 (1.85)
Personality disorders	22 (20.37)	1 (0.93)
Autism spectrum disorder	8 (7.40)	0 (0)
Dementia	2 (1.85)	0 (0)

*n* indicates the number of participants. *SD* Standard Deviation

### Psychosocial outcome measures

We selected a diverse range of psychosocial outcome measures that have been reported to be of relevance during the current pandemic [12, 28–31]. This includes mental health symptomatology measures of stress, anxiety, depression, and paranoia; transdiagnostic mental health factor measures of loneliness and rumination; and positive psychological functioning measures of psychological well-being and resilience.

#### Mental health symptomatology

##### Depression, Anxiety and Stress Scales-21 (DASS-21)

The German version of DASS-21 [32, 33] was used to measure anxiety, depression, and stress during the preceding week. Items are rated on a Likert scale of 0 (did not apply to me at all) to 3 (applied to me very much or most of the time). Higher scores indicate greater levels on each of the respective subscales. In clinical and non-clinical samples good psychometric properties of the scales have been reported [34]. In our study, DASS-21 subscale scores’ internal consistency ranged from good to excellent: *ω*_Anxiety_ = 0.84 (95% CI: 0.79, 0.88), *ω*_Depression_ = 0.93 (95% CI: 0.92, 0.95), and *ω*_Stress_ = 0.89 (95% CI: 0.86, 0.91).

##### Revised-Green et al. Paranoid Thoughts Scale (R-GPTS)

The total score of the German version of the 18-item R-GPTS [35, 36] that includes two subscales of ideas of reference (e.g., “People definitely laughed at me behind my back”) and ideas of persecution (e.g., “I was certain people did things in order to annoy me”) assessed over the past fortnight were used to measures paranoia. Items are rated on a 5-point Likert scale ranging from 0 (not at all) to 4 (totally). Scores can range from 0 to 72; higher scores indicate higher levels of paranoia. Excellent psychometric properties of the scales have been reported for the English version [36]. In our study, the R-GPTS subscale scores ranged from good to excellent with *ω*_Part A_ = 0.88 (95% CI: 0.85, 0.91) and *ω*_Part B_ = 0.91 (95% CI: 0.88, 0.94).

#### Transdiagnostic mental health factors

##### Perseverative Thinking Questionnaire (PTQ)

The PTQ [37] consists of 15 items and is a self-report scale, which measures content-independent negative ruminative thinking. Items are rated on a 5-point Likert scale ranging from 0 (Never) to 4 (Almost always). Higher scores indicate higher levels of ruminative thinking and scores can range from 0 to 60. Good psychometric properties have been reported in previous research [37]. In our study, the internal consistency of the PTQ was excellent *ω* = 0.97 (95% CI: 0.97, 0.98).

##### UCLA Loneliness Scale (UCLA)

The German version of the UCLA [38, 39] was used to assess loneliness. The intensity and frequency of feelings of loneliness are assessed with 20 items using a 5-point Likert scale ranging from 1 (not at all) to 5 (totally). Reversed items were recorded and then averaged to form a mean score, with higher scores indicating greater loneliness. The German version of the UCLA has been reported to show high internal consistency and discriminant validity [39]. We observed an excellent internal consistency with *ω* = 0.93 (95% CI: 0.91, 0.94).

#### Positive psychological functioning

##### Brief Resilience Scale (BRS)

The German version of the six items BRS [40, 41] was used to assess resilience. Items are rated on a 5-point Likert scale ranging from 1 (strongly disagree) to 5 (strongly agree). Reversed items were recoded to calculate mean scores with higher scores indicating greater resilience. Sound psychometric properties of the self-report questionnaire were reported in previous research [41]. In our study, internal consistency was good with *ω* = 0.88 (95% CI: 0.85, 0.91).

##### WHO (Five) Well-Being Index (WHO-5)

Participants were asked to complete the German version of the WHO-5 [42, 43] which assesses well-being over the past 2 weeks. Items are rated on a 6-point Likert scale ranging from 0 (not present) to 5 (constantly present). Scores are summed, with higher scores indicating greater well-being. Good psychometric properties have been reported in previous research [44]. We observed an excellent internal consistency with *ω* = 0.91 (95% CI: 0.89, 0.93).

### Statistical analyses

All analyses were conducted in *R* (v4.0.3; R Foundation for Statistical Computing) with packages *psych* (v1.8.12) [45], *lavaan* (v0.6-3.1295) [46], *careless* (v1.1.3) [47], *apaTables* (v2.0.5) [48], *MBESS* (v4.8.0) [49], and *missForest* [50].

#### Missing data

After conducting the different steps to ensure data integrity and quality (see Supplementary Fig. 1), we imputed missing values. Since we had continuous and categorical mixed-type data, missing data were handled by applying the non-parametric, iterative *MissForest* imputation, which is based on a random forest algorithm [50]. Out-of-bag (OOB) estimates per sample for the imputation error were OOB_PFC_ < 0.001 for the non-clinical and OOB_PFC_ = 0.153 for the clinical sample.

#### Descriptive statistics

First, internal consistency was calculated for the COVID-19-specific stressors index and all outcomes variables using McDonald's Omega [ω; 51] instead of Cronbach's α since assumptions are rarely met in practice [52; see “Measures”]. Descriptive statistics and the strength of statistical association between variables were tested using bivariate Pearson’s correlation coefficients, Chi-square tests (*χ*^*2*^), and unpaired two-sample *t* tests (Welch *t* test) when appropriate. We report magnitudes of effect sizes according to Cohen [53]: correlation coefficients of 0.10 are considered “small”, those of 0.30 are “medium”, and those of 0.50 are “large” with 95% CI using 5000 bootstrapped samples with replacement.

#### Multiple linear regression analyses

We ran multiple linear regression analyses to evaluate associations of case–control status, COVID-19-specific stressors and their interaction with mental health outcomes in the matched sample. These regression analyses were conducted unadjusted and adjusted for age, sex, and employment status. All independent variables were standardised to facilitate interpretation of regression coefficients (*β*s) and main effects. In an additional step, we repeated regression analyses using psychosocial outcome variables on their original scale and standardising these variables; results for outcome variables on original scales are presented in Tables and Figures and results for standardised outcome variables are presented in the Results section to facilitate comparison to other manuscripts and between scales, respectively. To assess the robustness of results, also against violations of homoscedasticity, we provide 95% bootstrapped CI using 5000 bootstrapped samples with replacement. All hypothesis testing was two-tailed according to α = 0.05. *R*^2^ is reported when appropriate.

#### Stratified analyses

To explore the respective impact of COVID-19-specific stressors on the different psychosocial outcome variables and in clinical and non-clinical samples separately, we performed additional group-stratified multiple regression analyses, again adjusted for age, sex, and employment status. For these analyses, both dependent and independent variables were standardised to allow effect size comparisons of the COVID-19-specific stressors predictor between samples and outcome variables.

#### Sensitivity analyses

To analyse the robustness and consistency of results, we applied four sets of sensitivity analyses. First, the same multiple linear regression analyses were repeated in the larger sample (*n* = 605) that was not matched on age, sex, and employment status, but also adjusted for these variables. Second, we repeated our primary analyses in the matched sample by excluding COVID-19-specific stressor items related to ‘living in a small accommodation’, ‘office work’, ‘customer service’, ‘childcare’, ‘running school lessons’, and ‘employment uncertainties’. This was done to explore consistency of results for those COVID-19-specific stressors that applied equally well to community-dwelling individuals and psychiatric inpatients and, thus, are of relevance across contexts. Third, multiple linear regression analyses were repeated in the matched sample while additionally adjusting for essential work activity for the maintenance of critical infrastructure (i.e., participants were grouped into the following categories (a) health care worker, (b) essential worker but non-healthcare worker, and (c) non-essential worker) and, finally, in separate analyses we controlled for the date of assessment using a linear and quadratic effect of time in addition to the matching variables.

## Results

### Descriptive statistics

Socio-demographics and baseline characteristics of clinical and non-clinical samples are displayed in Table 1 including self-reported life-time diagnoses. Based on clinician ratings in the psychiatric inpatient sample the majority of patients suffered from depression (77.14%), substance abuse disorders (49.52%), personality disorders (22.86%), and anxiety disorders (21.96%) with 76.85% of patients qualifying for > 1 psychiatric diagnosis based on the 10th of the International Statistical Classification of Diseases and related Health Problems (ICD-10) criteria (see Supplementary Table 1 for details).

**Fig. 1:**
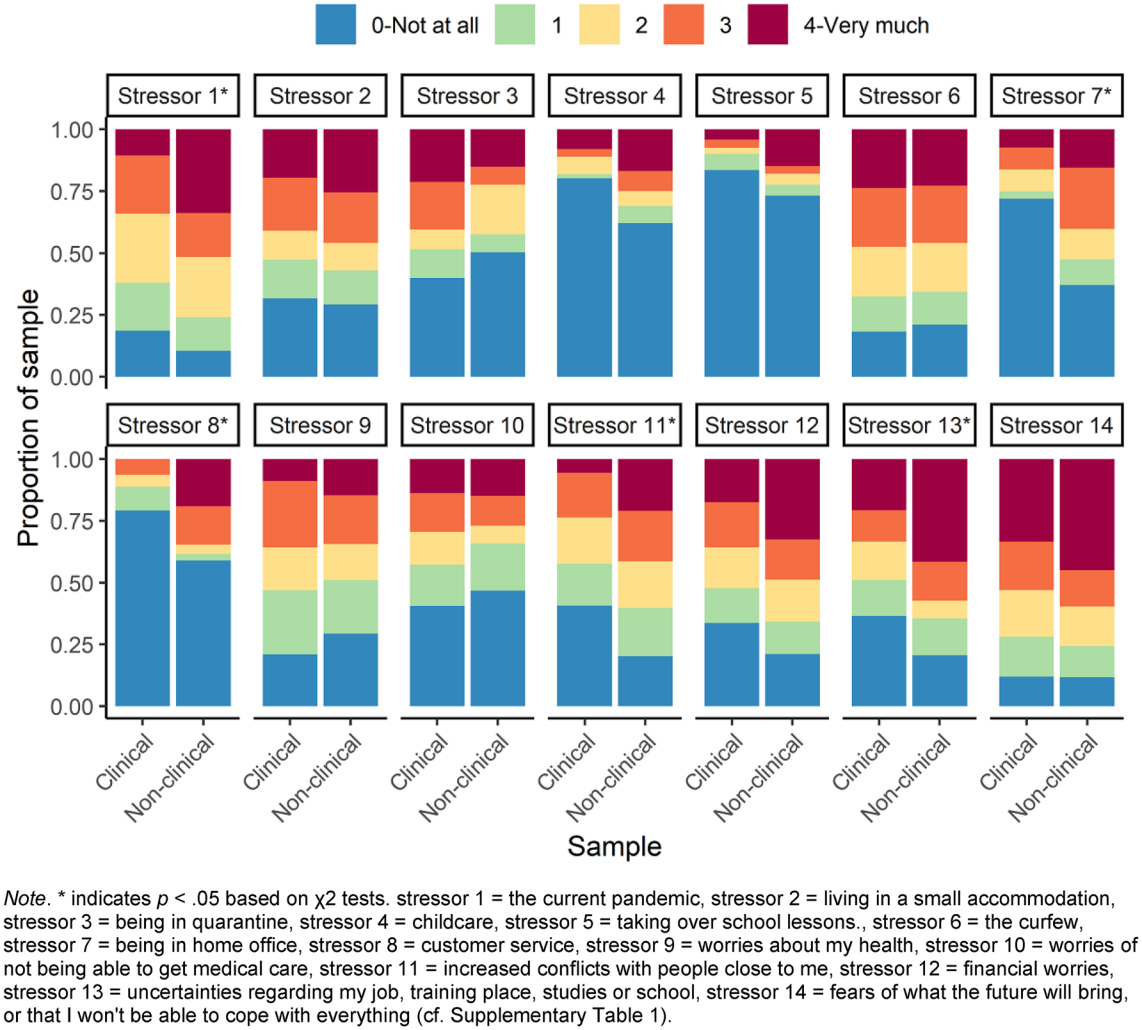
Comparison of the COVID-19-specific stressors in the matched samples

A comparison of COVID-19-specific stressors in the matched sample is shown in Fig. 1 (numeric results are presented in Supplementary Table 1). Overall, the total index score of COVID-19-specific stressors differed between groups (t(198.83) = 2.43, *p* < 0.016, Cohen’s *d* = 0.33), in that the non-clinical sample indicated a greater impact of COVID-19-specific stressors. Results comparing differences in COVID-19-specific stressors between the clinical and non-clinical samples show that the non-clinical sample had higher levels of stressors related to the current pandemic, home-office, customer service, interpersonal conflicts, and job uncertainties. For all other COVID-19-specific stressors such as quarantine/curfew, childcare responsibilities, and physical health concerns we did not observe evidence for differences between groups.

Table 2 includes descriptive statistics of outcome variables and results of Welch t-tests between groups on the different psychosocial outcomes. Results show that psychiatric inpatients displayed greater mental health difficulties as indicated by higher levels of anxiety, depression, stress, rumination, loneliness and lower levels of well-being and resilience, compared to non-clinical individuals. Effect sizes were observed to be medium to large (absolute Standardised Mean Difference (SMD) ranged from 0.47 to 0.98). We did not observe evidence for differences in paranoia between groups.

**Table 2:** Descriptive statistics and differences in psychosocial outcome variables between matched samples

Outcome	Clinical sample			Non-clinical sample			*p*	SMD	CI_bootstrappdSMD_
	Mean (SD)	Range	IQR	Mean (SD)	Range	IQR			
Anxiety (DASS-21)	12.61 (9.87)	0–40	5.50–18	6.30 (7.06)	0–28	0–10	< 0.001***	0.69	0.45, 0.91
Depression (DASS-21)	20.5 (13.05)	0–42	8–32	12.13 (11.6)	0–40	2–19	< 0.001***	0.64	0.40, 0.89
Stress (DASS-21)	18.46 (10.95)	0–42	10–26	13.5 (10.18)	0–40	5.5–20.5	< 0.001***	0.46	0.20, 0.71
Paranoia (R-GPTS)	10.5 (13.22)	0–61	1–14	9.15 (8.94)	0–38	2–13.3	0.38	0.12	− 0.15, 0.36
Rumination (PTQ)	35.53 (14.48)	0–60	25–46	25.69 (14.99)	0–58	13.8–37.3	< 0.001***	0.64	0.39, 0.87
Loneliness (UCLA)	2.63 (0.72)	1–4.6	2.2–3.1	2.15 (0.77)	1–4.5	1.5–2.6	< 0.001***	0.62	0.37, 0.85
Well-being (WHO-5)	7.12 (5.56)	0–22	3–11	12.62 (5.65)	1–23	8–17	< 0.001***	− 0.88	− 1.10, − 0.65
Resilience (BRS)	2.46 (0.76)	1–4.5	1–2.8	3.28 (0.99)	1–5	1–4.0	< 0.001***	− 0.84	− 1.06, − 0.62

***Indicates *p* < 0.001. SD is used to represent standard deviation. *IQR* inter quartile range. *P* values based on Welch two-sample *t* test. *SMD* Standardised Mean Difference. CI_bootstrappedSMD_ = 95% bootstrapped Confidence Interval of SMD

### Multiple linear regression analyses

**Fig. 2:**
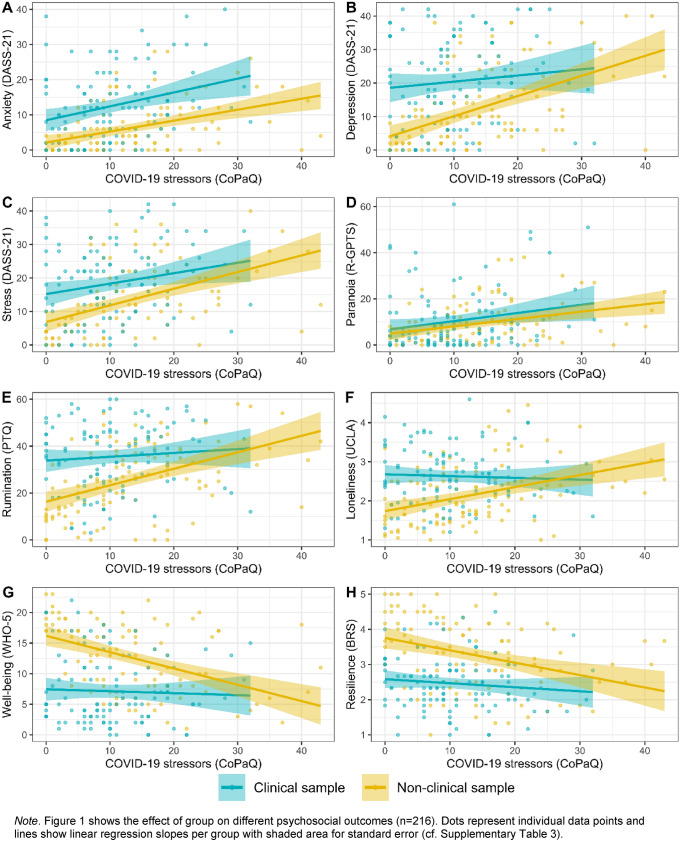
Associations of COVID-19-specific stressors with psychosocial outcomes in the matched samples

Results of the multiple linear regression analyses are depicted in Fig. 2 and numeric results are reported in Supplementary Table 3. Throughout multiple linear regression analyses, we observed significant associations of COVID-19-specific stressors with all psychosocial outcome variables including mental health symptomatology (increased levels of depression (standardised β[SE] = 0.27[0.06]), anxiety (standardised β = 0.34[0.06]), stress (standardised β = 0.33[0.07]), and paranoia (standardised β = 0.26[0.07])), transdiagnostic mental health factors (increased levels of rumination (standardised β = 0.25[0.06]) and loneliness (standardised β = 0.15[0.07])), and positive psychological functioning (less psychological well-being (standardised β = − 0.21[0.06]) and resilience (standardised β = − 0.21[0.06])). Group status was also significantly associated with all psychosocial outcome variables (depression (standardised β = 0.73[0.12]), anxiety (standardised β = 0.80[0.12]), stress (standardised β = 0.57[0.13]), rumination (standardised β = 0.72[0.12]), loneliness (standardised β = 0.66[0.13]), well-being (standardised β = − 0.95[0.12]), and resilience (standardised β = − 0.91[0.12])); paranoia (standardised β = 0.21[0.13]) was the only exception. For the psychosocial outcome variables depression (standardised β = − 0.28[0.13]), rumination (standardised β = − 0.31[0.13]), loneliness (standardised β = − 0.40[0.13]), and well-being (standardised β = 0.33[0.12]), we observed evidence for group by stressors interactions in the unadjusted model. These interactions unequivocally displayed a relatively greater increase of mental health difficulties in the non-clinical sample while mental health difficulties in the clinical sample were relatively stable across levels of COVID-19-specific stressors. No evidence for group by stressor interactions were observed for anxiety (standardised β = 0.08[0.13]), stress (standardised β = − 0.15[0.13]), paranoia (standardised β = 0.03[0.14]), and resilience (standardised β = 0.21[0.13]). When adjusting for age, sex, and employment status, findings remained substantially unchanged.

### Stratified analyses

**Fig. 3:**
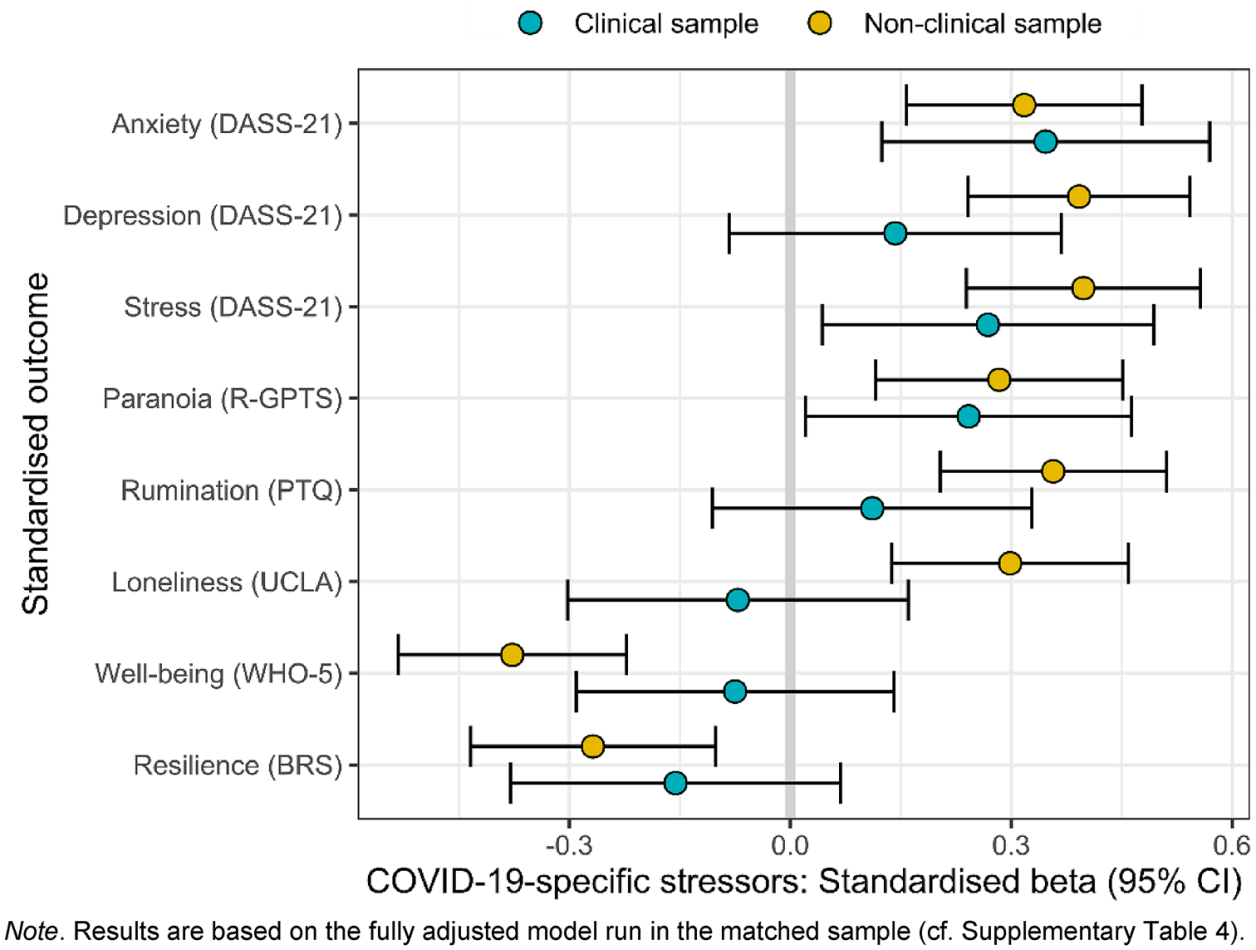
Patient status-stratified standardised associations of COVID-19-specific stressors with psychosocial outcomes

Figure 3 shows results of the patient-status stratified analyses with standardised dependent and independent variables and controlled for age, sex, and employment status (numeric results are displayed in Supplementary Table 4). In the non-clinical sample, we observed evidence of similarly strong associations between COVID-19-specific stressors and each psychosocial outcome (absolute beta coefficients ranged from 0.27 to 0.40). In contrast, in the clinical sample evidence for associations between COVID-19-specific stressors and depression, rumination, loneliness, well-being and resilience was either absent or negligible (absolute beta coefficients ranged from 0.07 to 0.16), while they were more similar to the non-clinical sample for anxiety, stress, and paranoia (absolute beta coefficients ranged from 0.24 to 0.35). These results also correspond to the findings of the multiple linear regression analyses.

### Sensitivity analyses

Results of the four sensitivity analyses are presented in Supplementary Tables 5–8. Briefly, results remained substantially unchanged in sensitivity analyses using the (1) unmatched sample, (2) reduced COVID-19-specific stressor index (interaction analyses of the group by stressors on depression, rumination, loneliness, and well-being appeared somewhat more robust and additional evidence for stress and resilience was observed), (3) additionally adjusting for essential work activities, and (4) controlling for date of assessment.

## Discussion

We followed the call by Holmes et al. [1] to assess the psychological response to the current pandemic by scrutinising the impact of COVID-19-specific stressors in vulnerable individuals with serious mental health disorders, compared to a matched sample of non-clinical controls. In line with the diathesis-stress-model [2] of mental disease, we hypothesised that the psychosocial impact of the pandemic is greatest in vulnerable individuals with severe mental health disorders. However, this hypothesis was not supported by our data. Instead, the impact of COVID-19-specific stressors was greater in non-clinical than in clinical respondents and these stressors were an important determinant for psychosocial functioning especially in the non-clinical sample. Importantly, psychiatric inpatients did not show a more adverse psychological response to stressors posed by the pandemic in terms of worse psychological functioning compared to non-clinical controls and, interestingly, COVID-19-specific stressors were consistently more strongly associated with depression, rumination, loneliness, and well-being in our non-clinical sample. This association followed a dose–response relationship, in which non-clinical individuals experiencing the greatest impact of COVID-19-specific stressors exhibited mental health symptomatology levels of psychiatric inpatients. Sensitivity analyses did not substantially change our results supporting their robustness.

Our cross-sectional findings on COVID-19-specific stressors add to previous longitudinal research showing an increase in mental health difficulties when comparing levels before versus during the early stages of the pandemic. This has been observed in studies of non-clinical individuals from a large UK general population sample [13, 18] and three Dutch psychiatric case–control cohort samples [17] with no additional mental health deterioration in vulnerable individuals with pre-pandemic mental health conditions [23]. Pan et al. [17] offer several explanations of these findings including mitigation strategy-induced relaxation, feelings of safety, or simply regression to the mean in vulnerable individuals with pre-existing mental disorders, whereas Pierce et al. [13] highlight the importance of tracking the longitudinal impact further into the pandemic. Our findings suggest that the impact of COVID-19-specific stressors could offer an additional explanation. In alignment with the diathesis-stress-model, we observed an increase in mental health difficulties in our non-clinical sample with increasing levels of COVID-19-specific stressors following a dose–response relationship.

Surprisingly, our findings indicated that psychiatric inpatients exhibited different patterns of associations of COVID-19-specific stressors with depression, rumination, loneliness, well-being and resilience as compared to anxiety and stress. Anxiety and stress are related constructs and DASS-21 anxiety and stress subscales entail items on physiological hyperarousal and psychological over-reactivity [54], which can be interpreted as the body’s and mind’s response to stress. These stress responses involving physiological hyperarousal and psychological over-reactivity seemed to be independent of psychiatric patient status in the present study. Contrary to this, the other psychosocial outcomes such as depression remained relatively unchanged in the presence of stressors in our psychiatric inpatient sample. Thus, we support Pan et al.’s explanations of mitigation strategy-induced relaxation and feelings of safety, which may be particularly enhanced in a psychiatric inpatient setting. That is, psychiatric inpatients, who are partly shielded from their external environment through the cover of hospitalisation, may be confronted less directly with the aversive consequences of the pandemic, compared to non-clinical individuals. This could also be exemplified by greater levels of COVID-19-specific stressors in the non-clinical group, which may ultimately result in psychological exhaustion. As such, replication of our results in psychiatric outpatient settings is key. Yet, Robinson et al. [23] in their recent systematic meta-analysis of population-based studies also find no deterioration of mental health symptomatology in those individuals with mental health conditions and propose that patients may generally be less exposed to stressors such as social interactions during the pandemic. Following this line of argument, it will be key to continue tracking symptom trajectories in this vulnerable group to see whether mental health difficulties will increase once the pandemic and associated countermeasures subside. Alternatively, psychiatric symptoms in distinct domains may have shown ceiling effects in the clinical sample, whereby depression, rumination, loneliness, well-being, and resilience were at their relative respective maximum or minimum. While distributions of the psychosocial outcomes do not fully support this explanation (Supplementary Fig. 3), this hypothesis requires further investigation. We additionally agree with the proposed characterisation of longitudinal trajectories in future research further into the pandemic [1], since the prolonged/chronic exposure to major stressors and strains caused by the pandemic could result in a “wear and tear” reflected in worse long-term mental health outcomes and, as our findings suggest, this may particularly affect non-clinical individuals [55].

### Strengths, limitations and future directions

Strengths of the present study include the examination of the psychological response to the pandemic based on (i) a wide array of key mental health measures, (ii) a large psychiatric inpatient sample, which is arguably one of the most vulnerable groups in terms of mental health difficulties, and (iii) use of a statistical matching procedure to a non-clinical group and a broad range of sensitivity analyses that supported the robustness of our results. This study has several important limitations. First and foremost, the study design is cross-sectional, which prevents causal interpretations. In particular, reverse causation or residual confounding cannot be excluded. For example, individuals with heightened anxiety levels may be more prone to experience a greater impact of COVID-19-specific stressors. Future longitudinal research is needed to assess directionality and evidence for temporality, which is one of Hill’s [56] viewpoints on causation. Second, we report data of convenience samples in that our non-clinical sample was predominantly female and younger than the general population (prior to matching) and the clinical sample represents a subset of patients from a single psychiatric hospital. While the generalisability of our findings is limited, the current study may have benefitted from the non-representativeness. The study was not set up to assess the prevalence of psychosocial difficulties but rather to identify in a case–control design whether stressors posed by the pandemic may differentially predict psychosocial difficulties. Therefore, the high number of individuals indicating psychosocial difficulties may have increased our statistical power to test these associations. However, replication of our results in more representative samples is key to determine the generalisability of our findings. By matching the clinical and non-clinical samples on age, sex, and employment status, we were able to mitigate these sample-dependent biases to some extent. However, the selection of less severely affected inpatients able to participate in a questionnaire-based study and exclusion of patients such as with acute psychosis and mania remains an important limitation of this study. Third, we report data of German samples, which limits cross-cultural generalizability. Fourth, we only relied upon self-report questionnaires. Finally, the items on COVID-19-specific stressor index may apply better to community-dwelling individuals than psychiatric inpatients. Yet, when applying sensitivity analyses with a reduced COVID-19-specific stressor index of stressors that apply equally well to both study groups, our results remained largely unchanged, which supports the robustness of our findings.

## Conclusions

Notwithstanding these caveats, our results may contribute to a better understanding of the mental health consequences of the current COVID-19 pandemic that could have both reassuring and concerning implications. On the one hand, we show that the psychological response to the pandemic is not worse in vulnerable individuals with serious mental health disorders compared to non-clinical individuals. On the other hand, our findings show that non-clinical individuals who experienced the greatest impact of COVID-19-specific stressors have levels of depression, rumination, loneliness, and well-being similar to psychiatric inpatients. These results have clinical and societal relevance suggesting that inpatient treatment efforts for patients with high levels of COVID-19-specific stressors should focus particularly on anxiety and stress symptomatology. Our results for non-clinical individuals could also help to identify individuals who were hit hardest by the pandemic and may be in need of targeted prevention and treatment efforts.

## Supplementary Information

Below is the link to the electronic supplementary material.

Supplementary file1 (DOCX 684 KB)
