# Supplementary material for: Differential psychological response to the COVID-19 pandemic in psychiatric inpatients compared to a non-clinical population from Germany
Source: Eur Arch Psychiatry Clin Neurosci. 2021 Jul 15;272(1):67–79. doi: 10.1007/s00406-021-01291-7 (PMC8282176; doi:10.1007/s00406-021-01291-7)
Supplement: Supplementary file 1 — Supplementary file1 (DOCX 684 KB) [file 406_2021_1291_MOESM1_ESM.docx]

ONLINE SUPPLEMENTARY MATERIAL

**Re: Differential Psychological Response to the COVID-19 Pandemic in Psychiatric Inpatients compared to a Non-Clinical Population from Germany**

Stephanie V. Rek, Daniel Freeman, Matthias A. Reinhard, Markus Bühner, Sofie Grosen, Peter Falkai, Kristina Adorjan, & Frank Padberg

**Table of Contents**

[SUPPLEMENTARY METHODS 3](#_Toc67564272)

[Data integrity and quality control 3](#_Toc67564273)

[Supplementary Figure 1. Data cleaning procedure 3](#_Toc67564274)

[SUPPLEMENTARY RESULTS 4](#_Toc67564275)

[Supplementary Figure 2. COVID-19-specific stressors item-intercorrelations in the matched sample 4](#_Toc67564276)

[Supplementary Figure 3. Distributions of the psychosocial outcomes in the clinical- and non-clinical sample 5](#_Toc67564277)

[Supplementary Table 1. Overview of response frequencies in (%) of the COVID-19-specific stressors in the matched samples 6](#_Toc67564278)

[Supplementary Table 2. Clinician’s ascertained psychiatric diagnoses in the psychiatric inpatient sample based on ICD-10 7](#_Toc67564279)

[Supplementary Table 3. Associations of COVID-19-specific stressors with psychosocial outcomes in unadjusted and adjusted multiple regression analyses in the matched samples 8](#_Toc67564280)

[Supplementary Table 4. Results from patient status stratified regression analyses of standardised psychosocial outcomes on standardised COVID-19-specific stressors in the matched samples 9](#_Toc67564281)

[Supplementary Table 5. Associations of COVID-19-specific stressors with psychosocial outcomes in unadjusted and adjusted multiple regression analyses in the unmatched samples 10](#_Toc67564282)

[Supplementary Table 6. Associations of the reduced COVID-19-specific stressors with psychosocial outcomes in unadjusted and adjusted multiple regression analyses in the matched samples 11](#_Toc67564283)

[Supplementary Table 7. Associations of the COVID-19-specific stressors with psychosocial outcomes in adjusted (sex, age, employment status, and essential work) multiple regression analyses in the matched samples 12](#_Toc67564284)

[Supplementary Table 8. Associations of the COVID-19-specific stressors with psychosocial outcomes in adjusted (sex, age, employment status, and time of assessment) multiple regression analyses in the matched samples 13](#_Toc67564285)

# SUPPLEMENTARY METHODS

Procedure

## Data integrity and quality control

Participants of both samples were excluded if they had more than 10% missing data on each of the self-report questionnaires or were identified as careless responders (their longest or average length of consecutive identical responses was ±3 SD of the respective sample mean). In the non-clinical online sample, we additionally excluded participants who gave incorrect responses on more than one out of three included bogus items (e.g., “Please, indicate completely agree”) and with response times <25 min, which we considered highly unlikely (median completion duration = 48 min).

## Supplementary Figure 1. Data cleaning procedure


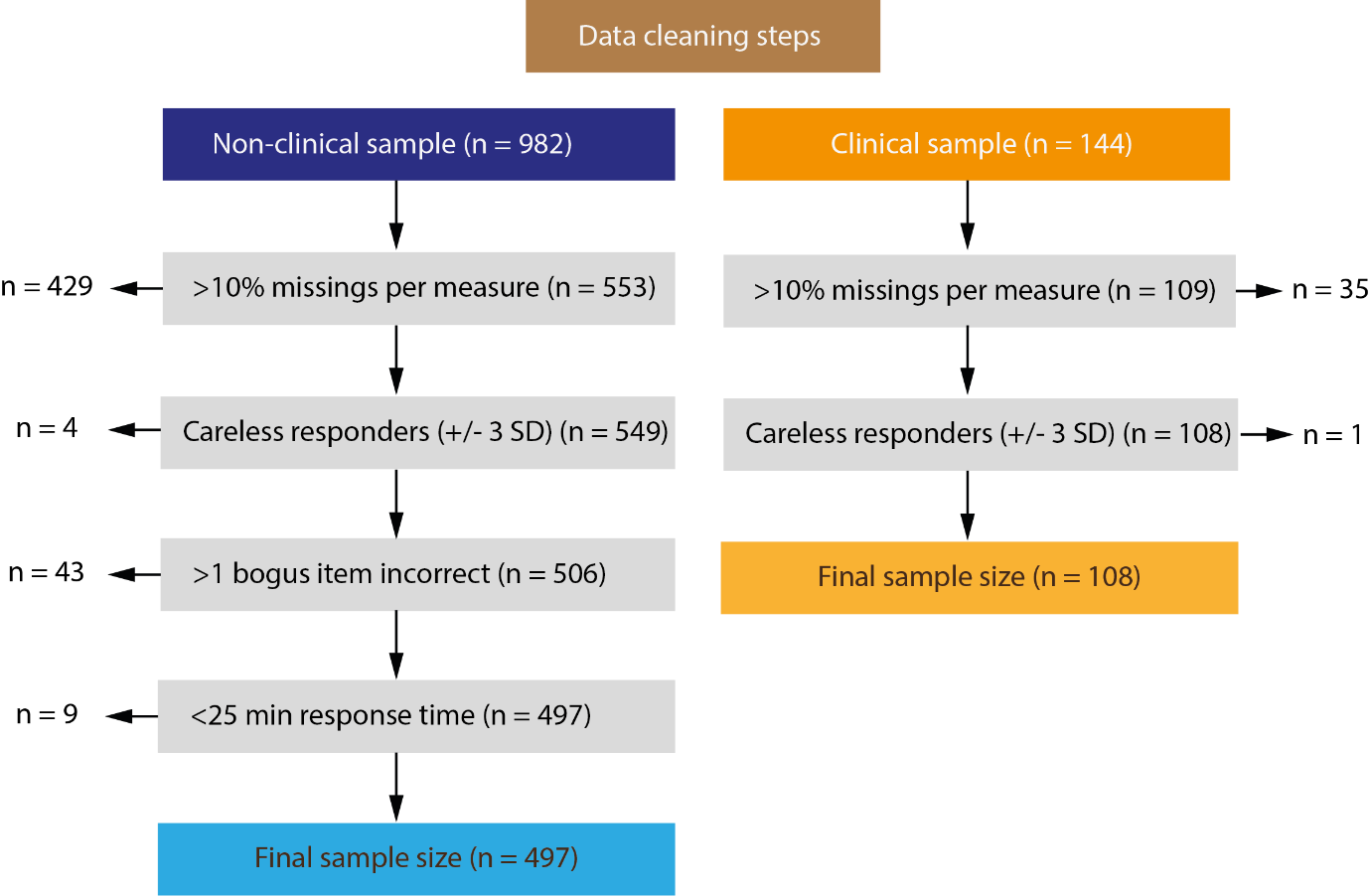


*Note*. Flowchart of study participants.

# SUPPLEMENTARY RESULTS

## Supplementary
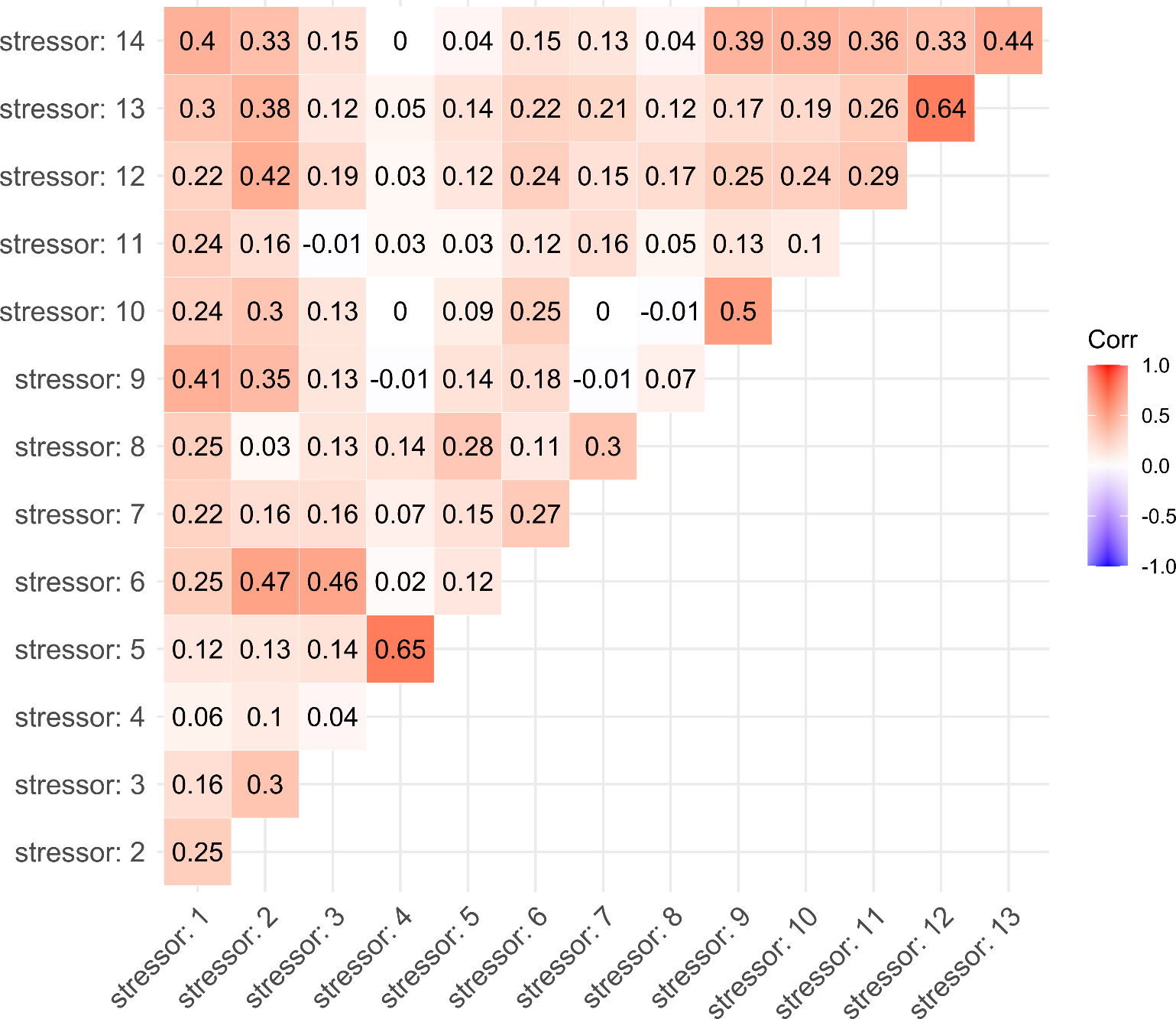
Figure 2. COVID-19-specific stressors item-intercorrelations in the matched sample

*Note*. stressor: 1 = the current pandemic, stressor: 2 = living in a small accommodation, stressor: 3 = being in quarantine, stressor: 4 = childcare, stressor: 5 = taking over school lessons., stressor: 6 = the curfew, stressor: 7 = being in home office, stressor: 8 = customer service, stressor: 9 = worries about my health, stressor: 10 = worries of not being able to get medical care, stressor: 11 = increased conflicts with people close to me, stressor: 12 = financial worries, stressor: 13 = uncertainties regarding my job, training place, studies or school, stressor: 14 = fears of what the future will bring, or that I won't be able to cope with everything.

## Supplementary Figure 3. Distributions of the psychosocial outcomes in the clinical- and non-clinical sample


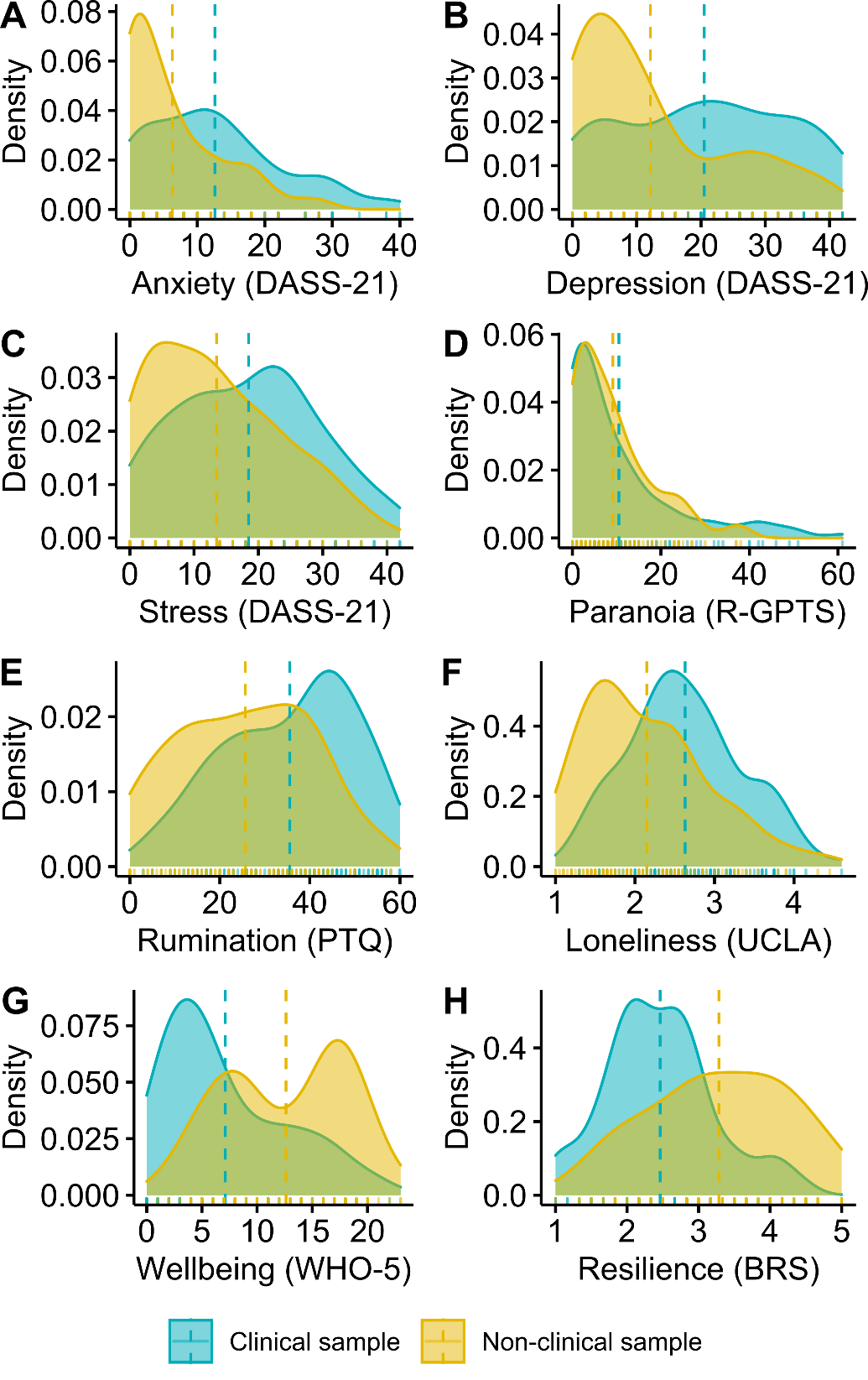


*Note*. Dashed lines represent respective sample means.

## Supplementary Table 1. Overview of response frequencies in (%) of the COVID-19-specific stressors in the matched samples

| “Because of the COVID-19 pandemic, **over the past 14 days** I have felt stressed or burdened a lot by…” | Non-clinical individuals (in %) \| Psychiatric Inpatient (in %) | | | | | |
| --- | --- | --- | --- | --- | --- | --- |
|  | 0  “Not at all” | 1 | 2 | 3 | 4  “Very much” | *p* |
| 1. the current pandemic. | 29 \| 41 | 19 \| 21 | 22 \| 20 | 12 \| 13 | 19 \| 5 | 0.021^*^ |
| 1. living in a small accommodation. | 58 \| 60 | 14 \| 15 | 7 \| 7 | 10 \| 10 | 10 \| 7 | 0.970 |
| 1. being in quarantine | 77 \| 69 | 6 \| 10 | 10 \| 5 | 3 \| 8 | 5 \| 7 | 0.098 |
| 1. childcare. | 85 \| 94 | 5 \| 1 | 3 \| 3 | 3 \| 1 | 5 \| 2 | 0.251 |
| 1. taking over school lessons. | 91 \| 94 | 3 \| 4 | 2 \| 1 | 1 \| 1 | 4 \| 1 | 0.677 |
| 1. the curfew. | 47 \| 43 | 15 \| 17 | 15 \| 16 | 13 \| 14 | 10 \| 11 | 0.975 |
| 1. being in home office. | 67 \| 90 | 9 \| 2 | 7 \| 4 | 11 \| 3 | 6 \| 2 | 0.001*** |
| 1. customer service. | 85 \| 91 | 2 \| 6 | 2 \| 2 | 6 \| 2 | 6 \| 0 | 0.037* |
| 1. worries about my health. | 56 \| 44 | 20 \| 27 | 9 \| 12 | 9 \| 14 | 6 \| 4 | 0.363 |
| 1. worries of not being able to get medical care. | 72 \| 68 | 15 \| 14 | 4 \| 7 | 5 \| 6 | 5 \| 5 | 0.761 |
| 1. increased conflicts with people close to me. | 44 \| 67 | 21 \| 14 | 14 \| 10 | 11 \| 7 | 9 \| 2 | 0.010** |
| 1. financial worries. | 48 \| 62 | 15 \| 13 | 13 \| 10 | 9 \| 8 | 15 \| 6 | 0.202 |
| 1. uncertainties regarding my job, training place, studies or school. | 48 \| 65 | 18 \| 13 | 6 \| 9 | 9 \| 6 | 19 \| 7 | 0.024* |
| 1. fears of what the future will bring, or that I won't be able to cope with everything. | 32 \| 31 | 18 \| 21 | 15 \| 17 | 10 \| 13 | 25 \| 18 | 0.687 |

*Note*. P-values are based on χ2 tests. * indicates *p* < .05. ** indicates *p* < .01. *** indicates *p* < .001.

## Supplementary Table 2. Clinician’s ascertained psychiatric diagnoses in the psychiatric inpatient sample based on ICD-10

| Clinician’s ascertained diagnoses | n (%) |
| --- | --- |
| Number of diagnoses |  |
| 0 | 0 (0) |
| 1 | 25 (23.15) |
| 2 | 43 (39.81) |
| 3 | 24 (22.22) |
| >= 4 | 16 (14.82) |
| Any diagnosis | 108 (100) |
| Diagnostic categories |  |
| Depressive Disorders (F32 - F34) | 81 (77.14) |
| Bipolar Disorders (F30, F31) | 11 (10.48) |
| Psychotic Disorders (F20, F22, F23, F25, F29) | 11 (10.48) |
| Anxiety Disorders (F40, F41) | 23 (21.91) |
| Post-Traumatic Stress Disorder (F43) | 19 (18.10) |
| Dissociative Disorders (F44) | 5 (4.76) |
| Obsessive-Compulsive and Related Disorders (F45) | 4 (3.81) |
| Eating Disorders (F50) | 5 (4.76) |
| Substance-Related and Addictive Disorders (F10 - F19) | 52 (49.52) |
| Attention-Deficit/Hyperactivity Disorder | 1 (0.95) |
| Somatoform Disorders (F45) | 6 (5.71) |
| Personality Disorders (F60, F61, F69) | 24 (22.86) |
| Autism Spectrum Disorder (F84) | 6 (5.71) |
| Organic Disorder (F06) | 3 (2.86) |
| Other (F70, F71, F79, F80, F84) | 5 (4.76) |

*Note*. n indicates the number of participants.

## Supplementary Table 3. Associations of COVID-19-specific stressors with psychosocial outcomes in unadjusted and adjusted multiple regression analyses in the matched samples

|  | Patient status | | | COVID-19-specific stressor | | | Interaction | | |  |
| --- | --- | --- | --- | --- | --- | --- | --- | --- | --- | --- |
| Outcome | β (SE) | CI_bootstrapped_ | p | β (SE) | CI_bootstrapped_ | p | β (SE) | CI_bootstrapped_ | p | R^2^ |
| ***Unadjusted*** |  |  |  |  |  |  |  |  |  |  |
| Anxiety | 7.33 (1.12) | 5.05, 9.56 | <0.001*** | 3.11 (0.58) | 1.78, 4.58 | <0.001*** | 0.74 (1.16) | -2.00, 3.60 | 0.527 | 0.23 |
| Depression | 9.50 (1.61) | 6.38, 12.56 | <0.001*** | 3.46 (0.84) | 1.64, 5.45 | <0.001*** | -3.69 (1.67) | -7.38, 0.39 | 0.029* | 0.21 |
| Stress | 6.13 (1.37) | 3.52, 8.73 | <0.001*** | 3.56 (0.71) | 2.07, 5.18 | <0.001*** | -1.64 (1.42) | -4.78, 1.60 | 0.251 | 0.18 |
| Paranoia | 2.32 (1.51) | -0.67, 5.31 | 0.126 | 2.97 (0.79) | 1.06, 4.99 | <0.001*** | 0.31 (1.58) | -3.68, 4.33 | 0.843 | 0.07 |
| Rumination | 11.09 (1.92) | 7.39, 14.86 | <0.001*** | 3.82 (1.00) | 1.88, 6.00 | <0.001*** | -4.76 (2.01) | -8.74, -0.55 | 0.019* | 0.20 |
| Loneliness | 0.52 (0.10) | 0.33, 0.71 | <0.001*** | 0.12 (0.05) | 0.02, 0.23 | 0.026* | -0.31 (0.10) | -0.51, -0.11 | 0.003** | 0.17 |
| Well-being | -5.93 (0.73) | -7.32, -4.55 | <0.001*** | -1.32 (0.38) | -2.16, -0.58 | <0.001*** | 2.07 (0.76) | 0.39, 3.58 | 0.007** | 0.28 |
| Resilience | -0.89 (0.12) | -1.11, -0.65 | <0.001*** | -0.21 (0.06) | -0.33, -0.09 | <0.001*** | 0.21 (0.12) | -0.01, 0.44 | 0.090 | 0.25 |
| ***Adjusted*** |  |  |  |  |  |  |  |  |  |  |
| Anxiety | 7 .21 (1.20) | 4.74, 9.52 | <0.001*** | 2.93 (0.59) | 1.60, 4.38 | <0.001*** | 0.98 (1.17) | -1.88, 3.81 | 0.404 | 0.26 |
| Depression | 8.40 (1.72) | 4.93, 11.52 | <0.001*** | 3.49 (0.85) | 1.65, 5.42 | <0.001*** | -3.36 (1.67) | -6.90, 0.47 | 0.046* | 0.25 |
| Stress | 5.95 (1.47) | 3.14, 8.66 | <0.001*** | 3.63 (0.72) | 2.05, 5.32 | <0.001*** | -1.36 (1.43) | -4.48, 1.75 | 0.343 | 0.21 |
| Paranoia | 2.19 (1.60) | -0.97, 5.32 | 0.174 | 2.79 (0.79) | 0.85, 4.67 | <0.001*** | 0.93 (1.56) | -3.19, 4.94 | 0.552 | 0.14 |
| Rumination | 10.02 (2.00) | 6.03, 13.72 | <0.001*** | 3.82 (0.98) | 1.87, 5.9 | <0.001*** | -4.27 (1.94) | -8.11, -0.14 | 0.029* | 0.29 |
| Loneliness | 0.49 (0.11) | 0.27, 0.7 | <0.001*** | 0.10 (0.05) | 0.00, 0.2 | 0.058 | -0.30 (0.10) | -0.50, -0.09 | 0.005** | 0.19 |
| Well-being | -5.46 (0.77) | -6.84, -4.03 | <0.001*** | -1.36 (0.38) | -2.18, -0.61 | <0.001*** | 1.83 (0.75) | 0.31, 3.34 | 0.015* | 0.35 |
| Resilience | -0.81 (0.12) | -1.04, -0.58 | <0.001*** | -0.21 (0.06) | -0.33, -0.09 | <0.001*** | 0.17 (0.12) | -0.04, 0.40 | 0.160 | 0.30 |

*Note*. Results are based on the matched sample and show the respective multiple regression models of the psychosocial outcome variables on standardised predictor variables for COVID-19-specific stressors, group (coded as -0.5 = non-clinical group, 0.5 = clinical group) and their interaction. CI_bootstrapped_ = 95% bootstrapped Confidence Interval. Intercept not shown. * indicates *p* < .05. ** indicates *p* < .01. *** indicates *p* < .001.

## Supplementary Table 4. Results from patient status stratified regression analyses of standardised psychosocial outcomes on standardised COVID-19-specific stressors in the matched samples

| Outcomes | Clinical sample | | Non-clinical sample | |
| --- | --- | --- | --- | --- |
|  | Beta (SE) | p | Beta (SE) | p |
| Anxiety (DASS-21) | 0.35 (0.11) | 0.003** | 0.32 (0.08) | <0.001*** |
| Depression (DASS-21) | 0.14 (0.11) | 0.218 | 0.39 (0.08) | <0.001*** |
| Stress (DASS-21) | 0.27 (0.11) | 0.021* | 0.40 (0.08) | <0.001*** |
| Paranoia (R-GPTS) | 0.24 (0.11) | 0.034* | 0.28 (0.09) | 0.001** |
| Rumination (PTQ) | 0.11 (0.11) | 0.317 | 0.36 (0.08) | <0.001*** |
| Loneliness (UCLA) | -0.07 (0.12) | 0.547 | 0.30 (0.08) | <0.001*** |
| Well-being (WHO-5) | -0.08 (0.11) | 0.497 | -0.38 (0.08) | <0.001*** |
| Resilience (BRS) | -0.16 (0.11) | 0.177 | -0.27 (0.08) | 0.002** |

*Note*. * indicates p < .05. ** indicates p < .01. *** indicates p < .001.

## Supplementary Table 5. Associations of COVID-19-specific stressors with psychosocial outcomes in unadjusted and adjusted multiple regression analyses in the unmatched samples

|  | Patient status | | | COVID-19-specific stressor | | | Interaction | | |  |
| --- | --- | --- | --- | --- | --- | --- | --- | --- | --- | --- |
| Outcome | β (SE) | CI_bootstrapped_ | p | β (SE) | CI_bootstrapped_ | p | β (SE) | CI_bootstrapped_ | p | R^2^ |
| ***Unadjusted*** |  |  |  |  |  |  |  |  |  |  |
| Anxiety | 7.36 (0.82) | 5.37, 9.40 | <0.001*** | 3.34 (0.46) | 2.09, 4.72 | <0.001*** | 0.42 (0.92) | -2.16, 3.17 | 0.649 | 0.23 |
| Depression | 9.48 (1.14) | 6.81, 12.19 | <0.001*** | 3.24 (0.64) | 1.63, 5.17 | <0.001*** | -3.17 (1.29) | -6.37, 0.68 | 0.014* | 0.23 |
| Stress | 6.12 (1.00) | 3.88, 8.42 | <0.001*** | 3.70 (0.56) | 2.32, 5.21 | <0.001*** | -1.79 (1.12) | -4.56, 1.10 | 0.111 | 0.22 |
| Paranoia | 1.31 (1.22) | -1.55, 4.23 | 0.283 | 3.52 (0.69) | 1.50, 5.54 | <0.001*** | -0.65 (1.38) | -4.81, 3.35 | 0.638 | 0.10 |
| Rumination | 8.98 (1.54) | 5.77, 12.06 | <0.001*** | 3.11 (0.87) | 1.37, 5.01 | <0.001*** | -3.26 (1.73) | -6.72, 0.49 | 0.060 | 0.14 |
| Loneliness | 0.47 (0.08) | 0.32, 0.63 | <0.001*** | 0.11 (0.04) | 0.02, 0.20 | 0.012* | -0.31 (0.09) | -0.49, -0.12 | <0.001*** | 0.15 |
| Well-being | -5.98 (0.57) | -7.09, -4.86 | <0.001*** | -1.27 (0.32) | -2.00, -0.63 | <0.001*** | 1.97 (0.64) | 0.51, 3.28 | 0.002** | 0.26 |
| Resilience | -0.85 (0.09) | -1.02, -0.68 | <0.001*** | -0.18 (0.05) | -0.28, -0.09 | <0.001*** | 0.16 (0.10) | -0.03, 0.35 | 0.105 | 0.20 |
| ***Adjusted*** |  |  |  |  |  |  |  |  |  |  |
| Anxiety | 7.70 (0.99) | 5.63, 10.03 | <0.001*** | 3.27 (0.46) | 1.95, 4.59 | <0.001*** | 0.49 (0.91) | -2.14, 3.15 | 0.594 | 0.26 |
| Depression | 8.44 (1.39) | 5.25, 11.75 | <0.001*** | 3.34 (0.64) | 1.63, 5.06 | <0.001*** | -3.02 (1.29) | -6.3, 0.60 | 0.019* | 0.25 |
| Stress | 6.49 (1.22) | 3.90, 9.07 | <0.001*** | 3.76 (0.56) | 2.34, 5.22 | <0.001*** | -1.56 (1.12) | -4.33, 1.21 | 0.165 | 0.24 |
| Paranoia | 0.79 (1.49) | -2.05, 4.41 | 0.595 | 3.50 (0.69) | 1.49, 5.44 | <0.001*** | -0.38 (1.37) | -4.34, 3.65 | 0.782 | 0.13 |
| Rumination | 9.97 (1.87) | 6.49, 13.76 | <0.001*** | 3.16 (0.86) | 1.50, 5.04 | <0.001*** | -2.81 (1.72) | -6.43, 0.95 | 0.103 | 0.17 |
| Loneliness | 0.39 (0.10) | 0.20, 0.59 | <0.001*** | 0.10 (0.04) | 0.01, 0.20 | 0.023* | -0.31 (0.09) | -0.49, -0.13 | <0.001*** | 0.18 |
| Well-being | -5.67 (0.69) | -6.99, -4.34 | <0.001*** | -1.33 (0.32) | -2.03, -0.66 | <0.001*** | 1.81 (0.63) | 0.37, 3.05 | 0.004** | 0.29 |
| Resilience | -0.80 (0.11) | -1.00, -0.58 | <0.001*** | -0.19 (0.05) | -0.28, -0.09 | <0.001*** | 0.13 (0.10) | -0.05, 0.31 | 0.180 | 0.25 |

*Note*. Results are based on the unmatched sample and show the respective multiple regression models of the psychosocial outcome variables on standardised predictor variables for COVID-19-specific stressors, group (coded as -0.5 = non-clinical group, 0.5 = clinical group) and their interaction. CI_bootstrapped_ = 95% bootstrapped Confidence Interval. Intercept not shown. * indicates *p* < .05. ** indicates *p* < .01. *** indicates *p* < .001.

## Supplementary Table 6. Associations of the reduced COVID-19-specific stressors with psychosocial outcomes in unadjusted and adjusted multiple regression analyses in the matched samples

|  | Patient status | | | COVID-19-specific stressor | | | Interaction | | |  |
| --- | --- | --- | --- | --- | --- | --- | --- | --- | --- | --- |
| Outcome | β (SE) | CI_bootstrapped_ | p | β (SE) | CI_bootstrapped_ | p | β (SE) | CI_bootstrapped_ | p | R^2^ |
| ***Unadjusted*** |  |  |  |  |  |  |  |  |  |  |
| Anxiety | 6.93 (1.11) | 4.66, 9.15 | <0.001*** | 2.94 (0.56) | 1.71, 4.26 | <0.001*** | -0.59 (1.12) | -3.07, 2.15 | 0.599 | 0.23 |
| Depression | 9.08 (1.57) | 6.08, 12.18 | <0.001*** | 3.40 (0.80) | 1.81, 5.22 | <0.001*** | -5.69 (1.59) | -8.79, -1.94 | <0.001*** | 0.24 |
| Stress | 5.72 (1.33) | 3.18, 8.34 | <0.001*** | 3.61 (0.68) | 2.23, 5.09 | <0.001*** | -3.44 (1.35) | -6.23, -0.41 | 0.012* | 0.21 |
| Paranoia | 1.90 (1.51) | -1.09, 4.83 | 0.209 | 2.63 (0.77) | 0.72, 4.55 | <0.001*** | -0.33 (1.53) | -4.42, 3.51 | 0.829 | 0.06 |
| Rumination | 10.61 (1.90) | 6.95, 14.39 | <0.001*** | 3.69 (0.97) | 1.83, 5.69 | <0.001*** | -5.99 (1.93) | -9.61, -2.10 | 0.002** | 0.21 |
| Loneliness | 0.51 (0.10) | 0.32, 0.69 | <0.001*** | 0.13 (0.05) | 0.03, 0.23 | 0.011** | -0.34 (0.10) | -0.53, -0.16 | <0.001*** | 0.18 |
| Well-being | -5.82 (0.71) | -7.19, -4.45 | <0.001*** | -1.54 (0.36) | -2.26, -0.89 | <0.001*** | 2.68 (0.72) | 1.15, 4.00 | <0.001*** | 0.32 |
| Resilience | -0.87 (0.12) | -1.09, -0.64 | <0.001*** | -0.21 (0.06) | -0.33, -0.10 | <0.001*** | 0.30 (0.12) | 0.08, 0.52 | 0.010* | 0.26 |
| ***Adjusted*** |  |  |  |  |  |  |  |  |  |  |
| Anxiety | 6.94 (1.20) | 4.52, 9.26 | <0.001*** | 2.71 (0.58) | 1.51, 4.04 | <0.001*** | -0.38 (1.14) | -2.93, 2.31 | 0.740 | 0.25 |
| Depression | 8.09 (1.69) | 4.68, 11.21 | <0.001*** | 3.39 (0.82) | 1.71, 5.18 | <0.001*** | -5.48 (1.60) | -8.59, -2.07 | <0.001*** | 0.27 |
| Stress | 5.70 (1.44) | 2.91, 8.45 | <0.001*** | 3.69 (0.70) | 2.20, 5.32 | <0.001*** | -3.24 (1.37) | -6.10, -0.27 | 0.019* | 0.24 |
| Paranoia | 1.86 (1.61) | -1.32, 4.96 | 0.250 | 2.33 (0.78) | 0.37, 4.13 | 0.003** | 0.43 (1.52) | -3.85, 4.38 | 0.776 | 0.12 |
| Rumination | 9.63 (1.99) | 5.61, 13.30 | <0.001*** | 3.61 (0.97) | 1.82, 5.50 | <0.001*** | -5.57 (1.88) | -8.99, -1.91 | 0.003** | 0.29 |
| Loneliness | 0.48 (0.11) | 0.27, 0.69 | <0.001*** | 0.11 (0.05) | 0.01, 0.20 | 0.043* | -0.33 (0.10) | -0.53, -0.15 | 0.001** | 0.20 |
| Well-being | -5.40 (0.75) | -6.73, -4.00 | <0.001*** | -1.54 (0.36) | -2.31, -0.84 | <0.001*** | 2.51 (0.71) | 1.10, 3.82 | <0.001*** | 0.38 |
| Resilience | -0.79 (0.12) | -1.02, -0.56 | <0.001*** | -0.20 (0.06) | -0.33, -0.09 | <0.001*** | 0.27 (0.12) | 0.06, 0.48 | 0.020* | 0.31 |

*Note*. Results are based on the matched sample and show the respective multiple regression models of the psychosocial outcome variables on standardised predictor variables for COVID-19-specific stressors, group (coded as -0.5 = non-clinical group, 0.5 = clinical group) and their interaction. CI_bootstrapped_ = 95% bootstrapped Confidence Interval. Intercept not shown. * indicates *p* < .05. ** indicates *p* < .01. *** indicates *p* < .001.

## Supplementary Table 7. Associations of the COVID-19-specific stressors with psychosocial outcomes in adjusted (sex, age, employment status, and essential work) multiple regression analyses in the matched samples

|  | Patient status | | | COVID-19-specific stressor | | | Interaction | | |  |
| --- | --- | --- | --- | --- | --- | --- | --- | --- | --- | --- |
| Outcome | β (SE) | CI_bootstrapped_ | p | β (SE) | CI_bootstrapped_ | p | β (SE) | CI_bootstrapped_ | p | R^2^ |
| ***Adjusted*** |  |  |  |  |  |  |  |  |  |  |
| Anxiety | 7.66 (1.21) | 5.23, 9.96 | <0.001*** | 2.87 (0.59) | 1.50, 4.30 | <0.001*** | 0.87 (1.16) | -1.96, 3.64 | 0.454 | 0.28 |
| Depression | 8.97 (1.70) | 5.54, 12.15 | <0.001*** | 3.2 (0.84) | 1.41, 5.06 | <0.001*** | -3.51 (1.63) | -7.03, 0.08 | 0.033* | 0.30 |
| Stress | 6.51 (1.46) | 3.66, 9.21 | <0.001*** | 3.43 (0.72) | 1.86, 5.10 | <0.001*** | -1.50 (1.40) | -4.68, 1.57 | 0.284 | 0.26 |
| Paranoia | 2.13 (1.63) | -1.10, 5.28 | 0.194 | 2.69 (0.80) | 0.67, 4.62 | <0.001*** | 0.94 (1.56) | -3.25, 4.99 | 0.548 | 0.14 |
| Rumination | 10.41 (2.01) | 6.33, 14.16 | <0.001*** | 3.57 (0.99) | 1.57, 5.67 | <0.001*** | -4.38 (1.93) | -8.17, -0.4 | 0.024* | 0.31 |
| Loneliness | 0.50 (0.11) | 0.29, 0.72 | <0.001*** | 0.09 (0.05) | -0.01, 0.19 | 0.088 | -0.3 (0.10) | -0.51, -0.1 | 0.004** | 0.20 |
| Well-being | -5.71 (0.77) | -7.12, -4.25 | <0.001*** | -1.31 (0.38) | -2.11, -0.58 | <0.001*** | 1.90 (0.74) | 0.43, 3.43 | 0.011* | 0.37 |
| Resilience | -0.82 (0.13) | -1.05, -0.57 | <0.001*** | -0.2 (0.06) | -0.33, -0.08 | 0.002** | 0.17 (0.12) | -0.04, 0.4 | 0.158 | 0.30 |

*Note*. Results are based on the matched sample and show the respective multiple regression models of the psychosocial outcome variables on standardised predictor variables for COVID-19-specific stressors, group (coded as -0.5 = non-clinical group, 0.5 = clinical group) and their interaction. CI_bootstrapped_ = 95% bootstrapped Confidence Interval. Intercept not shown. * indicates *p* < .05. ** indicates *p* < .01. *** indicates *p* < .001.

## Supplementary Table 8. Associations of the COVID-19-specific stressors with psychosocial outcomes in adjusted (sex, age, employment status, and time of assessment) multiple regression analyses in the matched samples

|  | Patient status | | | COVID-19-specific stressor | | | Interaction | | |  |
| --- | --- | --- | --- | --- | --- | --- | --- | --- | --- | --- |
| Outcome | β (SE) | CI_bootstrapped_ | p | β (SE) | CI_bootstrapped_ | p | β (SE) | CI_bootstrapped_ | p | R^2^ |
| ***Adjusted*** |  |  |  |  |  |  |  |  |  |  |
| Anxiety | 6.96 (1.23) | 4.73, 9.21 | <0.001*** | 3.19 (0.6) | 1.81, 4.67 | <0.001*** | 1.23 (1.18) | -1.67, 4.08 | 0.298 | 0.27 |
| Depression | 8.13 (1.76) | 5.01, 11.22 | <0.001*** | 3.83 (0.86) | 1.91, 5.93 | <0.001*** | -2.99 (1.69) | -6.75, 0.88 | 0.077 | 0.27 |
| Stress | 5.49 (1.48) | 2.71, 8.28 | <0.001*** | 4.1 (0.72) | 2.44, 5.82 | <0.001*** | -0.89 (1.42) | -4.15, 2.35 | 0.533 | 0.25 |
| Paranoia | 2.32 (1.65) | -0.69, 5.47 | 0.162 | 2.9 (0.81) | 0.85, 4.89 | <0.001*** | 1.13 (1.58) | -3.22, 5.33 | 0.476 | 0.14 |
| Rumination | 9.44 (2.05) | 5.58, 13.07 | <0.001*** | 4.19 (1) | 2.15, 6.34 | <0.001*** | -3.97 (1.96) | -8.02, 0.24 | 0.043* | 0.30 |
| Loneliness | 0.52 (0.11) | 0.32, 0.73 | <0.001*** | 0.11 (0.05) | 0.01, 0.22 | 0.044* | -0.27 (0.11) | -0.49, -0.06 | 0.011* | 0.20 |
| Well-being | -5.4 (0.79) | -6.86, -3.95 | <0.001*** | -1.49 (0.39) | -2.35, -0.7 | <0.001*** | 1.68 (0.75) | 0.04, 3.22 | 0.027* | 0.36 |
| Resilience | -0.79 (0.12) | -1.02, -0.56 | <0.001*** | -0.26 (0.06) | -0.37, -0.14 | <0.001*** | 0.11 (0.12) | -0.11, 0.34 | 0.351 | 0.35 |

*Note*. Results are based on the matched sample and show the respective multiple regression models of the psychosocial outcome variables on standardised predictor variables for COVID-19-specific stressors, group (coded as -0.5 = non-clinical group, 0.5 = clinical group) and their interaction. CI_bootstrapped_ = 95% bootstrapped Confidence Interval. Intercept not shown. * indicates *p* < .05. ** indicates *p* < .01. *** indicates *p* < .001.
